# Toxicological Study of *Ocimum sanctum* Linn Leaves: Hematological, Biochemical, and Histopathological Studies

**DOI:** 10.1155/2014/135654

**Published:** 2014-01-29

**Authors:** M. K. Gautam, R. K. Goel

**Affiliations:** Department of Pharmacology, Institute of Medical Sciences, Banaras Hindu University, Varanasi 221005, India

## Abstract

The present study was aimed to study the acute and subacute toxicity studies with orally administered 50% ethanolic leaves extract of *Ocimum sanctum* Linn (OSE). In acute toxicity tests, four groups of mice (*n* = 6/group/sex) were orally treated with doses of 200, 600, and 2000 mg/kg, and general behavior, adverse effects, and mortality were recorded for up to 14 days. In subacute toxicity study, rats received OSE by gavage at the doses of 200, 400, and 800 mg/kg/day (*n* = 6/group/sex) for 28 days, and biochemical, hematological, and histopathological changes in tissues (liver, kidney, spleen, heart, and testis/ovary) were determined. OSE did not produce any hazardous symptoms or death and CNS and ANS toxicities in the acute toxicity test. Subacute treatment with OSE did not show any change in body weight, food and water consumption, and hematological and biochemical profiles. In addition, no change was observed both in macroscopic and microscopic aspects of vital organs in rats. Our result showed that *Ocimum sanctum* extract could be safe for human use.

## 1. Introduction

Plants have always been an important source of drugs. A large number of the world's populations, especially in developing countries, depend upon medicinal plants as an alternative and complimentary drugs therapy for various ailments. Some of the most common practices involve the use of crude plant extracts, which may contain a broad diversity of molecules with often unknown biological effects [1]. Since the medicinal plants are being used indiscriminately without notifying to their possible unhealthy or toxic effects, the World Health Organization has recommended that traditional plants used for the treatment of diseases need further scientific investigation on their toxic side effects [2]. Plants produce bioactive compounds which act as defense mechanisms against any disease, and at the same time, may be toxic in nature [3]. However, the general acceptability of herbal medicines has been limited by a lack of defined chemical characterization, dose regimen, and adequate toxicity data to evaluate their safety [4]. Therefore, it has become essential to assess the safety of plants used for medicinal purposes for possible toxicity.

*Ocimum sanctum* Linn (Labiatae), known as holy basil, is a commonly used home remedy and has been advocated for various ailments like cold, fever, dysentery, hemorrhage and dyspepsia, glucoma, cataract, chronic conjunctivitis, and other painful eye diseases, as well as gastric and hepatic disorders in indigenous system of medicine [5]. The plant is endowed with a variety of pharmacological properties including antistress, antifertility, immunoregulatory, hypoglycemic, antibacterial, antifungal, antiinflammatory, anti-carcinogenic, antioxidant, and cyclooxygenase inhibitory [6]. OS has been reported to show gastroduodenal ulcer protective, antisecretory, and gastric mucosal defense enhancing activities [7]. The leaves of *O. sanctum* (OS) contain a volatile oil composed of limonene, borneol, copaene, caryophyllene, and elemol, phenolic compounds (rosmarinic acid, apigenin, cirsimaritin, and isothymusin), flavonoids (orientin and vicenin), and aromatic compounds (methyl chavicol and methyl eugenol) [6]. Eugenol forms the major active constituent of OS, even though other minor constituents like fixed oils and flavones have also been reported to have pharmacological activities [5].

As to the best of our knowledge, there is no reference about the safe dosage of *Ocimum sanctum* Linn in traditional medicine so it was thought worthwhile to do the acute toxicity (mortality and CNS/ANS toxicities) in mice and subacute toxicity (biochemical, hematological, and histopathological) in rats.

## 2. Materials and Methods

### 2.1. Experimental Animals

Inbred Charles-Foster albino rats (160–180 g) and Swiss albino mice (20–25 g) of either sex were obtained from the central animal house of the Institute of Medical Sciences, Banaras Hindu University, Varanasi. They were kept in the departmental animal house at 26 ± 20°C and relative humidity of 44–56%, with light and dark cycles of 10 and 14 h, respectively, for one week before and during the experiments. Animals were provided with standard rodent pellet diet (Pashu Aahar Vihar, Ramnagar, Varanasi) and water *ad libitum*. “Principles of laboratory animal care” (NIH publication number 82-23, revised 1985) guidelines were followed. Approval from the Central Animal Ethical Committee of the University was taken prior to the experimental work (Notification number-Dean/2010-11/173 dated 23.07.2010).

### 2.2. Plant Material and Preparation of Extract

The leaves of *Ocimum sanctum *(OS) (Ayurvedic Gardens, Banaras Hindu University) were collected during October-December and identified with the standard sample preserved in the Department of Dravyaguna, Institute of Medical Sciences, Banaras Hindu University, Varanasi. 50% ethanolic extract of OS (OSE) was prepared by adding 500 g of dried, crushed, and powdered leaves of OS in 1000 mL of 50% ethanol in a round bottom flask and was kept at room temperature for 3 days in shade. The extract was filtered and the above procedure was repeated twice. The extract filtrate so obtained was pooled and evaporated on water bath till it dried. The yield of OSE was about 5.00% (w/w).

### 2.3. Acute Toxicity Study

Adult Swiss albino mice of either sex, weighing between 20 and 25 g, fasted overnight and were used for acute toxicity study, as per the Organization for Economic Co-Operation and Development (OECD 423) guideline [8]. Four groups of mice (*n* = 6) of both sexes was fasted overnight. The first control group mice received 0.5% carboxymethyl cellulose (CMC) suspension in distilled water while the other three groups received OSE suspended in 0.5% CMC at doses of 200, 600, and 2000 mg/kg. The above doses were selected on the basis of our previous reported work on OSE where 400 mg/kg was found to have good ulcer protective effects [7]. Animals were observed closely for first 4 hours, for any toxicity manifestation, like increased motor activity, salivation, convulsion, coma, and death. Subsequently observations were made at regular intervals for 24 h. The animals were under further investigation up to a period of 14 days and the number of mice that died within the study period was noted [9].

### 2.4. Subacute Toxicity Study

The repeated doses (28 days) for oral toxicity studies were carried out in rats according to the OECD test guideline 407 [10]. Rats were divided randomly into 4 groups of 6 animals each (3 males and 3 females). After an overnight fast, animals in group I received 0.5% CMC suspension (control group), whereas rats in groups II, III, and IV (test groups) received OSE suspended in 0.5% CMC at the doses of 200, 400, and 800 mg/kg body weight, respectively. Doses of OSE and CMC were administered daily by oral gavage in the volume of 10 mL/kg body weight, once daily for 28 consecutive days. The animals were observed daily for any abnormal clinical signs and death during the study period. Body weights were measured and recorded at the beginning and then after every week of the experiment. At the end of the study, all animals fasted overnight (water *ad libitum*) and, on 29th day, the animals were weighed. Blood was collected from retroorbital technique with or without EDTA for haematological and biochemical analysis, respectively. The animals were sacrificed with an overdose of ether and other body organs were taken out for detailed weight and histopathological changes.

#### 2.4.1. Haematological Parameters

Haemoglobin, total leukocyte count, and differential leukocyte count (polymorph, lymphocyte, and eosinophil) [11] were determined in control and OSE-treated groups.

#### 2.4.2. Biochemical Estimations

The serum was carefully aspirated with a Pasteur pipette into sample bottles for the various biochemical assays. Assay kits (Span diagnostic reagent kit and Precichem diagnostic kit) were employed for aspartate transaminase (AST), alanine transaminase (ALT), alkaline phosphate (ALP), creatinine, blood glucose, total protein, and total cholesterol and bilirubin analysis was determined in the serum following the procedure described in the kits.

#### 2.4.3. Organs Weight and Histology

The rats were quickly dissected and the liver, kidneys, stomach, spleen, lung, heart, and testis or ovary were excised and weighed. The specimens for histopathology were fixed in 10% neutral, buffered formalin for 18 h at 4°C. 3-4 *μ*m in thickness of each specimen of liver, kidney, heart, spleen, and testis/ovary was cut and stained with hematoxylin and eosin stain following the standard laboratory procedures. The stained sections were examined under microscope for any cellular damage or change in morphology of that particular tissue.

### 2.5. Statistical Analysis

Statistical comparison was performed using one way analysis of variance (ANOVA) followed by Dunnett's test for multiple comparisons. All statistical analysis was performed using SPSS statistical version 16.0 software package (SPSS Inc., USA).

## 3. Result

### 3.1. Acute Toxicity Study

The limit dose of 2000 mg/kg did not cause death or any toxic signs in treated male and female mice. All six mice were normal throughout the study and survived until the end of the 14-day experiment period.

### 3.2. Subacute Toxicity Study

#### 3.2.1. General Observations

Oral administration of OSE at doses of 200, 400, or 800 mg/kg body weight daily for 28 day, did not produce any mortality. All the treated and control mice were normal throughout the study. The animals did not show any changes in general behavior or other physiological activities.

#### 3.2.2. Physical Parameters

Little or no change was observed in body weight, food consumption, and water intake in OSE (200, 400, and 800 mg/kg)-treated groups compared with control group after 28 days of study period in rats (Tables 1 and 2).

#### 3.2.3. Hematological Studies

Hematological parameters like mean haemoglobin content, WBC, RBC, and differential cell counts were not significantly different with OSE-treated rats from control group (Table 3).

#### 3.2.4. Biochemical Analysis

Biochemical parameters for liver and kidney function test like aspartate transaminase (AST), alanine transaminase (ALT), alkaline phosphatase (ALP), creatinine, albumin, blood glucose, total protein, total cholesterol, and bilirubin did not show any difference with the above doses of OSE compared to control group (Table 3).

#### 3.2.5. Organs Weight and Histology

**Figure 1:**
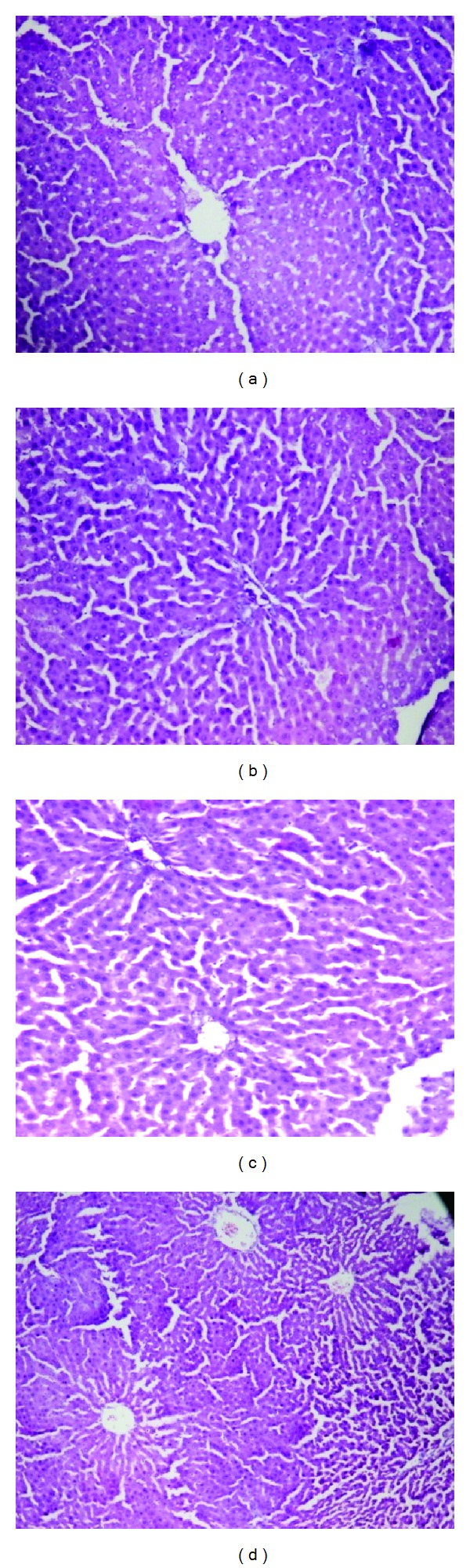
Histology of liver (H&E, 100x) of control and OSE-treated animals. (a) Section of liver from control animals revealed normal architecture and hepatic cells with granulated cytoplasm; ((b), (c), and (d)) liver from OSE (200, 400, and 800 mg/kg)-treated animals exhibited normal architecture of hepatocytes and hepatic cells with granulated cytoplasm.

**Figure 2:**
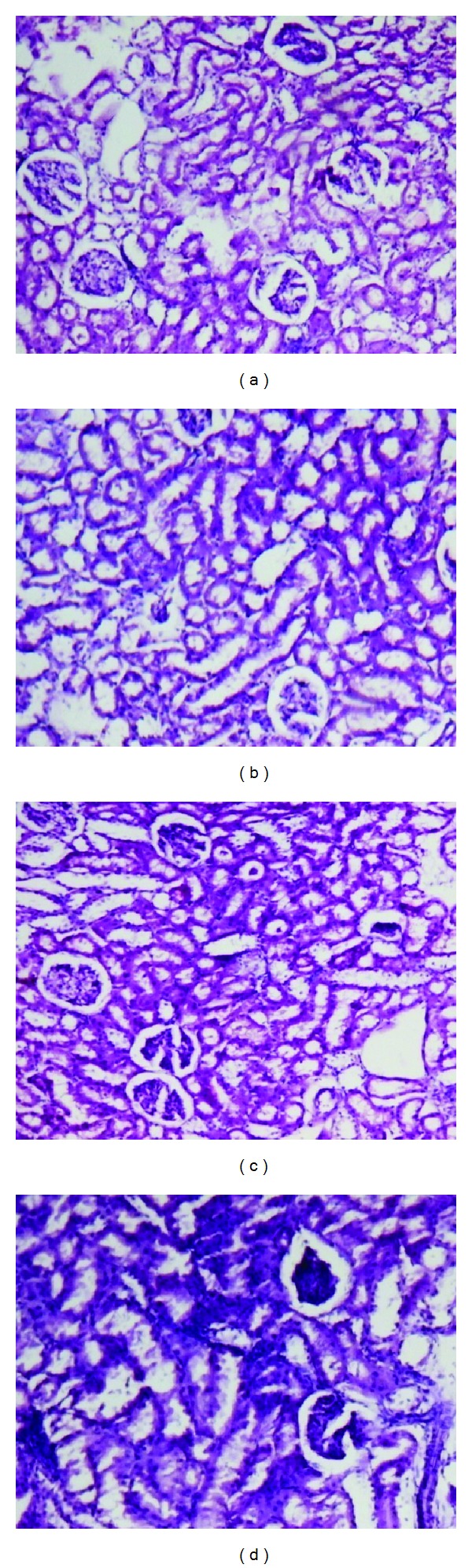
Histology of kidney (H&E, 100x) of control and OSE-treated animals. (a) Section of kidney from control animal showed normal size of glomeruli with normal tubules; ((b), (c), and (d)) kidney from OSE (200, 400, and 800 mg/kg)-treated animals exhibit normal size of glomeruli with normal tubules.

**Figure 3:**
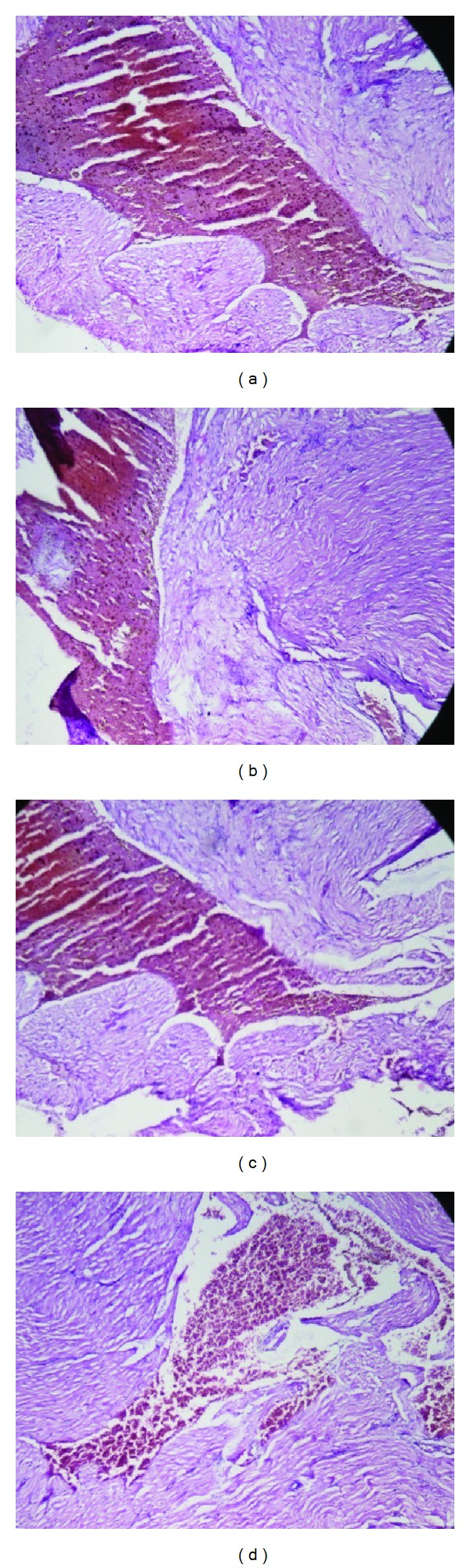
Histology of heart (H&E, 100x) of control and OSE-treated animals. (a) Section of heart from control animal showed normal muscle fibers with acidophilic cytoplasm and centrally located nuclei; ((b), (c), and (d)) heart from OSE (200, 400, and 800 mg/kg)-treated animals exhibit normal muscle fibers with acidophilic cytoplasm and centrally located nuclei.

**Figure 4:**
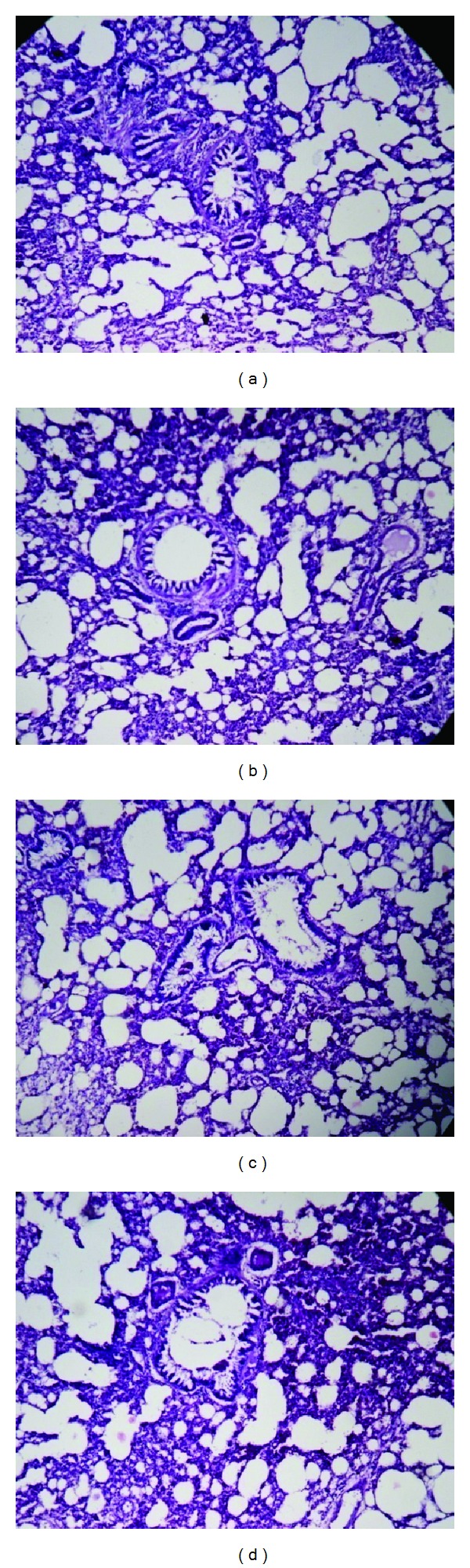
Histology of spleen (H&E, 100x) of control and OSE-treated animals. (a) Section of spleen from control animal showed normal granular hemosiderin pigment predominantly within macrophages in the red pulp; ((b), (c), and (d)) spleen from OSE (200, 400, and 800 mg/kg)-treated animals exhibit normal hemosiderin pigment predominantly within macrophages in the red pulp with normal structure.

**Figure 5:**
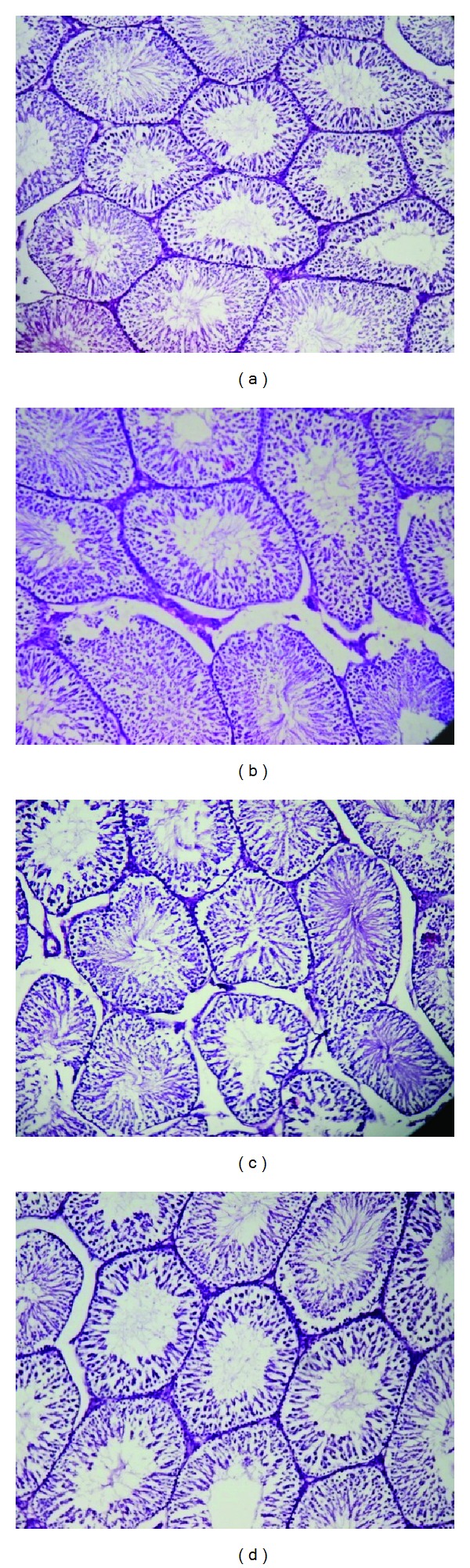
Histology of testis (H&E, 100x) of control and OSE-treated animals. (a) Section of testis from control animal showed well-layered seminiferous tubules with germ cell; ((b), (c), and (d)) testis from OSE (200, 400, and 800 mg/kg)-treated animals exhibit normal seminiferous tubules with germ cell.

**Figure 6:**
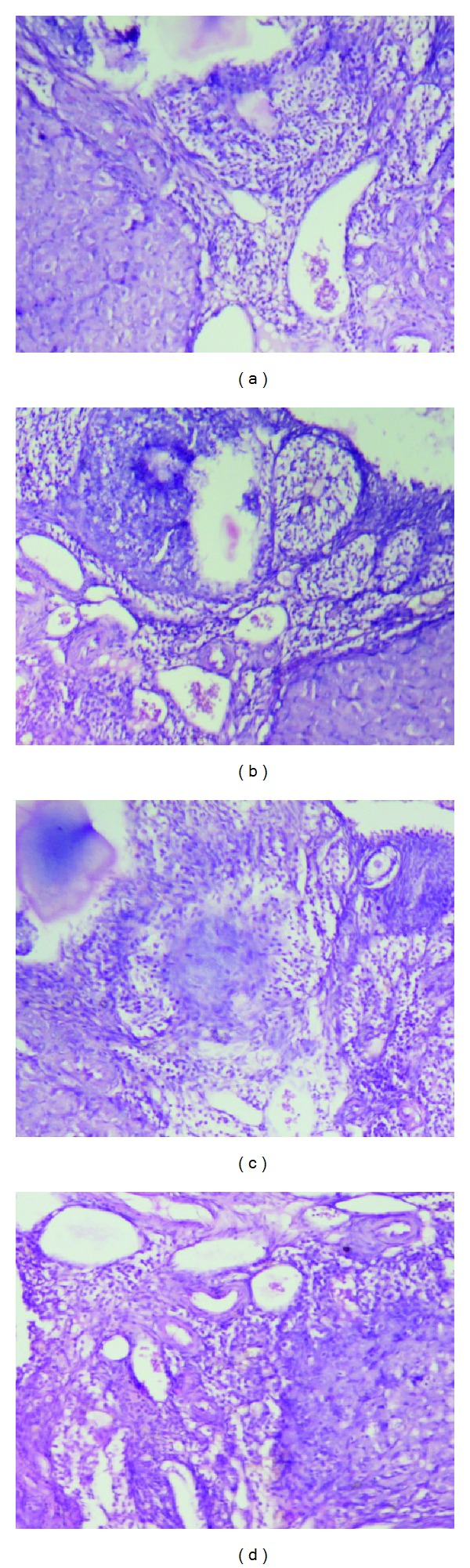
Histological of ovary (H&E, 100x) of control and experimental group of animals. (a) Section of ovary from control animal showed normal small follicles and large follicles; ((b), (c) and (d)) ovary from OSE 200, 400, and 800 mg/kg treated exhibit normal small follicles and large follicles in the histology.

The organs like liver, kidney, heart, spleen, and testis or ovary isolated in various groups did not reveal any abnormalities in their gross examinations and difference in their mean weights both in treated and control groups (Table 4). The histological studies with liver, spleen, kidney, heart, and testis/ovary did not reveal any pathological changes after treatment even with higher dose of 800 mg dose of OSE when administered for 28 days (Figures 1, 2, 3, 4, 5, and 6).

## 4. Discussion 

The administration of herbal preparations without any standard dosage, coupled with a scarcity of adequate scientific studies on their safety, has raised concerns regarding their toxicity [12]. Recently we reported teratological effects of *Asparagus racemosus* in rats which have been advocated in indigenous system of medicine during pregnancy and lactation [13], which thus indicated that herbal drugs are not safe as thought otherwise. To the best of our knowledge, there are no published studies on *Ocimum sanctum* toxicological profile following subacute exposure. In oral acute toxicity study, as high dose of OSE at 2000 mg/kg did not show any observable toxic effects in the mice in terms of any deaths or abnormal symptoms which points to its being nontoxic and safe in mice. Subacute toxicity study in rats with 200, 400, and 800 mg/kg of OSE when administered for 28 days did not produce any mortality, change in food and water intake, body and organ weights, or histopathological changes in the organs like liver, kidney, spleen, heart, testis or ovary further strengthen the safety profile of OSE.

Subacute studies in rats did not show any change in hematological, liver functions and spleen with 800 mg/kg OSE when administered for 28 days. The hematopoietic system/bone marrow is one of the most sensitive targets for toxic compounds and an important index of physiological and pathological status in man and animal [14]. Analysis of blood parameters is relevant to risk evaluation as the changes in the hematological system have a higher predictive value for human toxicity, when the data are translated from animal studies [15]. Subacute exposure of rat to the lower doses of the OSE produced small and transient changes in some biochemical and hematological parameters.

Liver is the major site for metabolism including drugs. Liver is a site of cholesterol disposal or degradation and its major site of synthesis. In the same perspective, it controls glucose synthesis and generates free glucose from hepatic glycogen stores [16]. Since no significant changes were observed in glucose and cholesterol levels this study, it suggests that OSE had no effect on the lipid and carbohydrate metabolism in rats. Further, drugs showing any toxicity in liver affect the transaminases, (aspartate aminotransferase) AST, and (alanine amino transferase) ALT which are well-known enzymes used as good indicators of liver function [17] and biomarkers predicting possible toxicity [18]. Generally, any damage to the parenchymal liver cells results in elevations of both transaminases in the blood [16]. In our study, both AST and ALT did not show any treatment related increase even at the 800 mg/kg dose compared to the control group. In addition, AST found in the serum is of both mitochondrial and cytoplasmic origin and any rise can be taken as a first sign of cell damage that leads to the outflow of the enzymes into the serum [19]. Thus, no significant increases observed in ALT and AST activities strongly suggest that the subacute administration of OSE did not alter the hepatocytes and consequently the metabolism in the rats. Further, OSE did not show any histological changes in spleen and liver indicating no effect on reticuloendothelial system.

OSE neither showed any significant difference in the weight of the organs or color of organs nor affected the histopathological changes in organs like heart and testis/ovary indicating least cumulative toxic effects on reproductive and cardiac tissues. Further, no change was also observed in kidney function as evidenced by little or no change in serum creatinine level as well as histological changes in treated kidneys.

## 5. Conclusion 

This study provides valuable data on the acute and subacute oral toxicities profile of *Ocimum sanctum* Linn leaves (a common household remedy for number of ailments) that could be very useful in its future clinical study. The 50% ethanol extract of *Ocimum sanctum* leaves seemed to be nontoxic as was seen after its acute and subacute oral administrations. Further, teratogenic, mutagenic and carcinogenic studies with this plant are needed to complete the safety profile of this plant.

## Figures and Tables

**Table 1:** Effect on body weight after 28 days oral administration of OSE.

Oral treatment (mg/kg, od)	Body weight (g)					
	0 day	3 days	7 days	14 days	21 days	28 days
Control 0.5% CMC	162.5 ± 6.34	166.3 ± 5.32	173.4 ± 5.46	185.0 ± 5.09	199.4 ± 5.78	215.0 ± 5.00
OSE 200	164.4 ± 6.01	165.0 ± 6.12	171.3 ± 4.20	186.9 ± 3.53	203.1 ± 7.49	220.0 ± 4.72
OSE 400	160.6 ± 5.38	162.5 ± 4.22	171.2 ± 6.39	188.8 ± 3.75	206.3 ± 5.95	222.6 ± 5.21
OSE 800	163.1 ± 5.58	166.3 ± 4.97	173.8 ± 3.75	190.3 ± 3.71	204.4 ± 3.46	223.8 ± 2.45

Values are expressed as mean ± SEM of 6 rats in each group.

**Table 2:** Effect on food intake and water intake after 28 days of the oral administration of OSE.

Oral treatment (mg/kg, od)	Food intake (g/day)						Water intake (mL/day)					
	0 day	3 days	7 days	14 days	21 days	28 days	0 day	3 days	7 days	14 days	21 days	28 days
Control 0.5% CMC	11.4 ± 0.70	11.63 ± 0.55	12.2 ± 0.61	12.9 ± 0.66	13.9 ± 0.79	15.2 ± 0.90	12.3 ± 0.46	12.5 ± 0.62	13.1 ± 0.74	14.2 ± 0.63	14.6 ± 0.76	15.5 ± 0.99
OSE 200	11.5 ± 0.77	12.2 ± 0.69	12.7 ± 0.59	13.8 ± 0.76	14.5 ± 0.67	15.6 ± 0.82	13.1 ± 0.5	13.7 ± 0.58	14.1 ± 0.76	14.9 ± 0.74	16.0 ± 0.85	16.4 ± 0.91
OSE 400	11.6 ± 0.63	12.1 ± 0.49	13.0 ± 0.57	13.7 ± 0.65	14.5 ± 0.78	15.9 ± 0.86	12.9 ± 0.55	13.7 ± 0.65	14.1 ± 0.67	15.2 ± 0.77	16.1 ± 0.91	17.2 ± 0.98
OSE 800	10.9 ± 0.27	11.4 ± 0.44	13.1 ± 0.77	13.3 ± 0.99	14.5 ± 0.94	15.2 ± 1.10	13.3 ± 0.53	13.6 ± 0.60	14.3 ± 0.72	15.2 ± 0.76	16.1 ± 0.83	16.1 ± 0.95

Values are expressed as mean ± SEM of 6 rats in each group.

**Table 3:** Effect on hematological and biochemical parameters after 28 days of the oral administration of OSE.

Parameters	Control group	OSE 200 mg/kg	OSE 400 mg/kg	OSE 800 mg/kg
RBC (million/mm^3^)	9.2 ± 1.1	9.1 ± 1.1	8.9 ± 0.8	9.1 ± 1.0
Hb (g/dL)	12.2 ± 1.2	12.2 ± 1.1	12.3 ± 0.7	12.1 ± 0.9
WBC (million/mm^3^)	8.8 ± 0.9	8.7 ± 1.2	8.9 ± 1.4	9.0 ± 1.1
Neutrophils %	22.35 ± 6.3	23.38 ± 9.3	22.58 ± 5.4	23.51 ± 8.7
Eosinophils %	2.37 ± 1.0	2.34 ± 1.3	2.82 ± 1.1	2.47 ± 1.4
Basophils %	0.18 ± 0.06	0.18 ± 0.08	0.15 ± 0.09	0.19 ± 0.06
Lymphocytes %	76.33 ± 2.7	74.46 ± 3.4	75.56 ± 2.5	73.35 ± 3.1
Monocytes %	3.38 ± 0.61	3.87 ± 0.72	4.04 ± 0.65	3.26 ± 0.73
AST (U/L)	195.90 ± 1.30	194.98 ± 1.90	196.85 ± 1.87	197.87 ± 1.08
ALT (U/L)	81.46 ± 1.23	80.72 ± 1.03	81.17 ± 2.07	83.5 ± 2.23
ALP (U/L)	232.28 ± 1.28	231.14 ± 1.60	232.26 ± 1.52	231.08 ± 1.07
Creatinine (mg/dL)	0.91 ± 0.07	0.90 ± 0.04	0.92 ± 0.02	0.92 ± 0.05
Albumin (g/dL)	2.84 ± 0.05	2.74 ± 0.07	2.83 ± 0.09	2.84 ± 0.08
Total protein (g/dL)	7.3 ± 1.3	7.2 ± 1.4	6.9 ± 1.5	7.1 ± 1.5
Glucose (mg/dL)	94.86 ± 4.63	89.53 ± 5.34	90.68 ± 6.39	91.53 ± 4.87
Total cholesterol (mg/dL)	122.8 ± 3.6	121.2 ± 4.4	118.9 ± 2.8	121.1 ± 3.9
Bilirubin total (mg/dL)	1.26 ± 0.21	1.23 ± 0.22	1.22 ± 0.31	1.21 ± 0.41
Bilirubin direct (mg/dL)	0.71 ± 0.02	0.68 ± 0.01	0.72 ± 0.01	0.70 ± 0.01

Values are expressed as mean ± SEM of 6 rats in each group.

**Table 4:** Effect on isolated organs weight after 28 days of the oral administration of OSE.

Oral treatment (mg/kg, od)	Isolated organs weight/100 g body weight rat					
	Liver	Right kidney	Heart	Spleen	Testis	Ovary
Control 0.5% CMC	2.7 ± 0.09	0.338 ± 0.01	0.360 ± 0.03	0.327 ± 0.01	0.649 ± 0.26	0.265 ± 0.17
OSE 200	2.6 ± 0.07	0.345 ± 0.02	0.354 ± 0.02	0.319 ± 0.01	0.668 ± 0.27	0.257 ± 0.20
OSE 400	2.7 ± 0.12	0.343 ± 0.01	0.352 ± 0.00	0.326 ± 0.02	0.656 ± 0.28	0.261 ± 0.16
OSE 800	2.7 ± 0.16	0.319 ± 0.01	0.364 ± 0.02	0.320 ± 0.01	0.661 ± 0.28	0.263 ± 0.18

Values are expressed as mean ± SEM of 6 rats in each group.

## Abbreviations

CNS: Central nervous system
ANS: Autonomous nervous system
RBC: Red blood cell
Hb: Hemoglobin
WBC: White blood cell
AST: Aspartate transaminase
ALT: Alanine transaminase
ALP: Alkaline phosphate.
